# Contribution of artificial intelligence to the imaging diagnosis of pediatric pulmonary tuberculosis

**DOI:** 10.1590/S1678-9946202668005

**Published:** 2026-01-30

**Authors:** Roberta Feijó Carvalho, Sandra Valéria Coelho da Silva, Michely Alexandrino de Souza Pinheiro, Rafaela Baroni Aurilio, Edwin Tao Ming Klinkenberg, Sara Vegas Viedma, Maria de Fátima Bazhuni Pombo Sant’Anna, Ana Alice Amaral Ibiapina Parente, Claudete Aparecida Araújo Cardoso, Clemax Couto Sant’Anna

**Affiliations:** 1 Universidade Federal do Rio de Janeiro Instituto de Puericultura e Pediatria Martagão Gesteira Rio de Janeiro Brazil Universidade Federal do Rio de Janeiro, Instituto de Puericultura e Pediatria Martagão Gesteira, Rio de Janeiro, Rio de Janeiro, Brazil; 2 Delft Imaging Systems B.V. Hertogenbosch Netherlands Delft Imaging Systems B.V., Hertogenbosch, The Netherlands; 3 Universidade Federal Fluminense Faculdade de Medicina Rio de Janeiro Brazil Universidade Federal Fluminense, Faculdade de Medicina, Niterói, Rio de Janeiro, Brazil

**Keywords:** Pulmonary tuberculosis, Children, Adolescents, Artificial intelligence, Chest radiograph, Computer-aided diagnosis

## Abstract

Pediatric tuberculosis (TB) remains a diagnostic challenge in Brazil and worldwide. The Brazilian Ministry of Health recommends a clinical scoring system (S-MoH) for children and adolescents with suspected TB. Interpretation of radiographs within this scoring system may require specialist input. AI-based systems, such as CAD4TB (Delft Imaging Systems B.V.), approved by the WHO for adults, are not yet recommended for standalone use in children under 15 years of age. A retrospective study was conducted at a pediatric institute from January 31, 2017, to January 29, 2025, including 179 patients aged 0–14 years with pulmonary TB or other diseases. CAD4TBv7.1 analyzed chest radiographs using two cutoff points established by Youden's index: 53.48 for analyses against the S-MoH score and 53.89 for analyses against microbiological confirmation. Results were compared with both microbiological confirmation and S-MoH score. Among the 179 participants, 61 (34.1%) had TB, 25 of which were microbiologically confirmed. CAD4TBv7.1 showed an area under the ROC curve (AUROC) of 0.71, with a sensitivity of 52% and a specificity of 86.3% compared with microbiological diagnosis. Against S-MoH, AUROC was 0.59, with a sensitivity of 34.43% and a specificity of 86.44%. CAD4TBv7.1 demonstrated low sensitivity and high specificity, particularly regarding its overall discriminative capacity. Thus, CAD4TBv7.1 emerges as a promising complementary screening tool for pediatric TB. Although its standalone use is not yet recommended, it may complement S-MoH in settings lacking radiologists. Investments in AI must be accompanied by consistent pediatric validation and strategies that combine technological innovation with traditional and cost-effective clinical approach.

## INTRODUCTION

Tuberculosis (TB) remains a significant public health issue in Brazil and worldwide. In 2024, Brazil reported 84,308 new cases of the disease, of which 4.1% (n=3,468) occurred in children and adolescents up to 15 years of age, and 2.4% (n=1,983) in children under 10 years old^1^.

The End TB Strategy, launched by the World Health Organization (WHO), aims to eliminate TB by 2035. A key component of this initiative is the early diagnosis of TB and the development of new diagnostic and screening methods^2^.

Due to diagnostic challenges of pulmonary TB (PTB) in the pediatric population, the Brazilian Ministry of Health (MoH) recommends using a scoring system for all children with suspected PTB and for adolescents with negative smear microscopy or Xpert MTB/RIF results (S-MoH). This score includes clinical and radiological findings, history of contact with an adult with TB, results of the tuberculin skin test (TST) or IGRA, and nutritional status^3,4^, classifying the likelihood of TB diagnosis into three categories: very likely (≥40 points), possible (30–35 points), and unlikely (≤25 points)^3,5^. Chest radiography is commonly used to guide clinical decision-making in pediatric TB, especially when microbiological tests are negative or unavailable. However, its usefulness is limited by inter-reader variability and the need for specialist interpretation^3,6^.

In 2021, the WHO approved the use of computer-aided detection (CAD) for TB screening programs targeting individuals over 15 years of age^6^. In June 2025, the WHO approved six software solutions for CAD use in TB screening. However, the organization does not yet recommend its use for screening children and adolescents under 15 years of age^7^.

CAD4TB (version 7.1, Delft Imaging Systems B.V., Hertogenbosch, The Netherlands) is an artificial intelligence (AI) based system that analyzes chest radiographs and generates a score from 0 to 100, indicating the probability that a patient has active TB. It also produces a heatmap highlighting lung regions considered abnormal. CAD4TB has demonstrated high accuracy in adults; however, results in children remains limited. The performance of CAD4TB version 7.1has been satisfactory in ruling out TB-incompatible cases, but its accuracy in detecting confirmed TB cases in children remains limited, highlighting the need for age-specific adjustments and validation^8^. Given this context, this study aims to assess the accuracy of CAD4TB version 7.1 in children and adolescents.

### Ethics

The study was approved by the Research Ethics Committee of Instituto de Puericultura e Pediatria Martagao Gesteira, Universidade Federal do Rio de Janeiro (IPPMG/UFRJ), under CAAE 81837724.1.0000.5264. All participants and their legal guardians signed an informed consent form. Patients aged seven to 14 years who were aware of their health status also signed an informed assent form to participate in the study.

## MATERIALS AND METHODS

A retrospective study was conducted at Instituto de Puericultura e Pediatria Martagao Gesteira, Universidade Federal do Rio de Janeiro (IPPMG/UFRJ), involving patients aged zero to 14 years diagnosed with either PTB or non-PTB diseases, between January 31, 2017, and January 29, 2025.

All patients with presumed PTB were consecutively included, defined as those with clinical, radiological, and/or microbiological data (Gene Xpert MTB/RIF Ultra® [Cepheid, USA] and/or culture) compatible with TB, according to the institution's medical assessment. The final TB diagnosis (confirmed or unconfirmed, according to Graham *et al.*^9^) was established after two months of follow-up. When TB was ruled out, participants were included in the comparison group (non-TB).

CAD4TBv7.1 generates a score ranging from 0 to 100, indicating the probability that a chest radiograph is compatible with TB. For screening purposes, the system adopts predefined thresholds: scores ≥30 correspond to high sensitivity, ≥50 to an intermediate threshold, and ≥70 to high specificity. In this study, two cut-off points were established using Youden's index^10^, according to the reference standard applied. For analyses against the microbiological standard, a cut-off of 53.89 was adopted, whereas for analyses against the S-MoH clinical score, a cut-off of 53.48 was used. Radiographs with scores equal to or above these thresholds were classified as suggestive of TB.

For statistical analysis assessing the diagnostic accuracy of CAD4TBv7.1, comparisons were made against two different reference standards: microbiologically confirmed TB diagnosis and the S-MoH clinical score. Patients classified as having TB according to the S-MoH were those with a score of 30 or higher, which corresponds to possible TB (30–35 points) or very likely TB (≥40 points).

In this study, the radiological component of the S-MoH score was defined according to the official criteria established by the MoH. Chest X-rays were interpreted at the time of clinical care by pediatricians and pulmonologists, and the findings recorded in medical charts were incorporated into the score as established. According to these criteria, chest radiographs showing hilar adenomegaly, miliary patterns, or consolidation/infiltrate (with or without cavitation) persisting for two weeks or more without improvement after antibiotic therapy for common pathogens were assigned 15 points. Radiographs with any type of consolidation or infiltrate lasting less than two weeks were assigned five points, while normal chest radiographs received −5 points. These radiological parameters were considered alongside other clinical and laboratory components of the scoring system^3,5^.

## RESULTS

A total of 179 patients were included, of whom 92 (51.4%) were female and 87 (48.6%) were male. Among the participants, 61 (34.1%) were diagnosed with TB, of which 25 (41%) were microbiologically confirmed (Gene Xpert MTB/RIF and/or culture).

**Figure 1:**
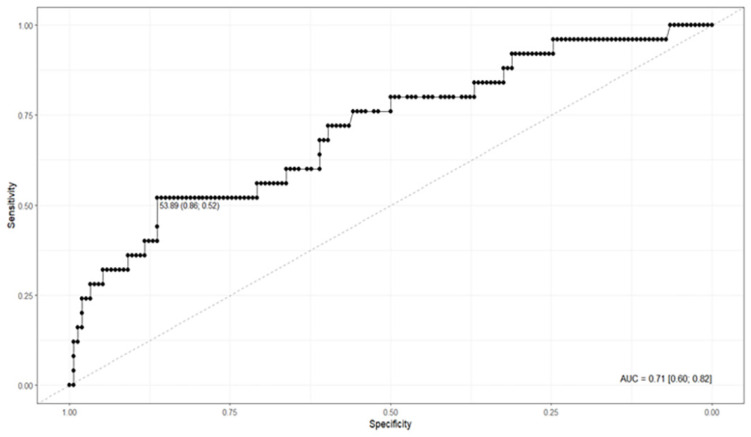
ROC Curve – Confirmed_TB and TB_score_CAD4TB_v7.1.

When comparing CAD4TBv7.1 to microbiologically confirmed TB, the tool demonstrated an area under the ROC curve (AUROC) of 0.71 (95%CI: 0.60–0.82), indicating moderate discriminative ability between positive and negative cases. Figure 1 shows a cut-off point of 53.89 and an area under the curve (AUC) of 0.71.

Sensitivity was 52.0%, specificity 86.36%, and overall accuracy 81.56%, with the cut-off point identified at 53.89. The analysis also showed a high negative predictive value (NPV) of 91.72%, suggesting good capacity to rule out the disease, although the positive predictive value (PPV) was relatively low (38.24%).

**Figure 2:**
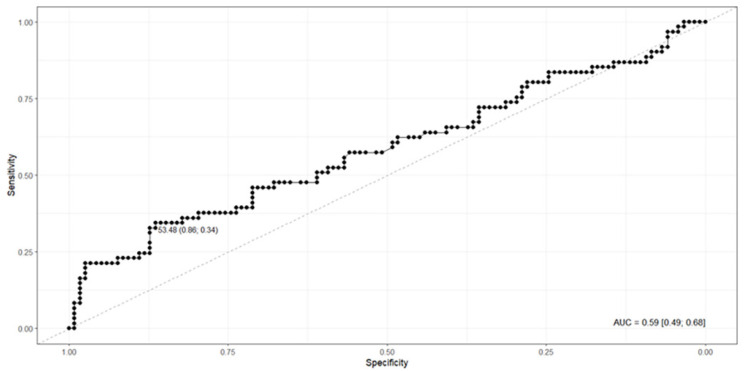
ROC Curve – TB Diagnosis according to SP-MS versus TB_score_CAD4TB v.7.1.

When compared to the diagnosis based on the SP-MS score, CAD4TBv7.1 showed an AUROC of 0.59 (95%CI: 0.49–0.68), sensitivity of 34.43%, specificity of 86.44%, and accuracy of 68.72%, with a cut-off point of 53.48. In this scenario, the predictive values demonstrated moderate performance, with an NPV of 71.83% and a PPV of 56.76%. Figure 2 shows a cut-off point of 53.89 and an AUC of 0.59. Detailed diagnostic performance metrics for both reference standards are summarized in Table 1.

**Table 1:** Diagnostic performance of CAD4TBv7.1 using two reference standards: S-MoH clinical score and microbiological confirmation.

Metric	Diagnosis_TB S-MoH (Youden = 53.48)	TB_confirmed (Youden = 53.89)
AUC	0.5864 (95% CI: 0.4934–0.6794)	0.7119 (95% CI: 0.5963–0.8276)
Threshold (Youden)	53.48	53.89
Specificity	0.864 (95% CI: 0.797–0.924)	0.864 (95% CI: 0.805–0.916)
Sensitivity	0.344 (95% CI: 0.230–0.475)	0.520 (95% CI: 0.320–0.720)
Accuracy	0.687 (95% CI: 0.631–0.743)	0.816 (95% CI: 0.760–0.866)
Negative predictive value (NPV)	0.718 (95% CI: 0.682–0.762)	0.917 (95% CI: 0.887–0.948)
Positive predictive value (PPV)	0.568 (95% CI: 0.432–0.719)	0.382 (95% CI: 0.258–0.517)

## DISCUSSION

Compared to the S-MoH, CAD4TBv7.1 demonstrated low sensitivity (34.43%) and high specificity (86.44%), particularly regarding its overall discriminative capacity (AUROC 0.59; 95%CI: 0.49–0.68). In our study, two cut-off points were defined according to the selected reference standard. This approach is widely applied in ROC curve analysis, as it provides a balance between correctly identifying true cases (sensitivity) and true non-cases (specificity). For the diagnosis_TB outcome, based on the S-MoH, the cut-off value of 53.48 resulted in a specificity of 86.4% and a sensitivity of 34.4%, with an overall accuracy of 68.7%. For the TB_confirmed outcome, based on microbiological confirmation, the cut-off value of 53.89 maintained the same specificity (86.4%) but showed higher sensitivity (52.0%) and an overall accuracy of 81.6%.

This pattern of high specificity combined with limited sensitivity is consistent with findings from other pediatric studies. Edem *et al*.^8^ reported a sensitivity of only 19% and a specificity of 99% in Gambian children, underscoring the challenge of achieving an adequate triage performance in this age group. Similarly, Palmer *et al*.^6^, in their evaluation of CAD4TB in South African children, observed poor initial performance (AUC 0.58), which improved to an AUC of 0.72 after fine-tuning with pediatric data and to 0.78 when only confirmed TB cases were considered. Despite this improvement, sensitivity remained below the WHO's minimum target profile for a triage test, confirming that the tool's performance remains suboptimal in pediatric cohorts. These results reinforce the need for validating cut-off points specifically adapted to the clinical and epidemiological context of pediatric populations.

In our study, Youden-derived thresholds (53.48 against the S-MoH and 53.89 against microbiological confirmation) yielded AUROC values of 0.59 and 0.71, respectively—both associated with restricted sensitivity but high specificity. These findings, aligned with those of Edem *et al*.^8^ and Palmer *et al*.^6^, reinforce the need for pediatric-specific cut-off validation and optimization strategies adapted to the clinical and epidemiological context of childhood TB. Thus, CAD4TBv7.1 emerges as a promising complementary screening tool for pediatric TB. Its use may be particularly valuable in settings lacking specialized radiologists or in large-scale screening programs, provided it is integrated into established clinical frameworks such as the S-MoH. Incorporating CAD4TBv7.1 as a support tool for clinical decision-making has the potential to optimize the diagnostic process and enhance early disease detection, provided that its results are interpreted with caution—especially in pediatric populations, for which system performance still requires specific validation.

To our knowledge, data on CAD performance in children remain limited, and several challenges remain, including the broader radiological spectrum observed in pediatric populations compared to adults. Pediatric chest anatomy varies across age groups and includes radiological features rarely observed in adults, such as enlargement of intrathoracic lymph nodes, which may cause airway narrowing or deviation without accompanying parenchymal disease. Studies have shown that, with fine-tuning training, the analysis of chest radiographs in children with suspected TB can be significantly improved^6^.

Comparisons between methods reveal complementary approaches. The S-MoH integrates clinical features, is tailored to local contexts, and is cost-effective. Diagnosis based on this system relies on clinical follow-up to assess treatment response. Conversely, CAD4TBv7.1 offers automation but requires digital infrastructure and still demands further validation in pediatric TB. Furthermore, the tool does not fulfill the performance requirements expected of a triage test in pediatric populations. At the Youden-derived thresholds (53.48 against the S-MoH and 53.89 against microbiological confirmation), it demonstrated high specificity but limited sensitivity; thus, its most appropriate role is as a decision-support tool integrated into clinical workflows—complementing established methods such as the S-MoH—rather than functioning as a stand-alone screening or rule-out test.

This study has certain limitations that should be considered. As a retrospective analysis based on secondary data, it was not possible to ensure full standardization of radiographic images or acquisition criteria throughout the study period. Additionally, lateral chest radiographs were not evaluated, which may have limited the scope of the analysis and negatively impacted the diagnostic accuracy for pulmonary TB.

## CONCLUSION

CAD systems should not fully replace comprehensive screening algorithms, as they cannot capture the clinical presentation and symptoms essential for pediatric TB diagnosis. However, CAD can support radiological assessments, and integrating tools such as the S-MoH score into CAD platforms could enhance their practical applicability. Investments in AI must be accompanied by consistent pediatric validation and strategies that combine technological innovation with traditional and cost-effective clinical approaches.

## Data Availability

The complete anonymized dataset supporting the findings of this study is available from https://doi.org/10.48331/SCIELODATA.NLQHXE
